# Screening of genetic alterations related to non-syndromic hearing loss using MassARRAY iPLEX® technology

**DOI:** 10.1186/s12881-015-0232-8

**Published:** 2015-09-23

**Authors:** Maria Carolina CCosta CMelo Svidnicki, Sueli Matilde Silva-Costa, Priscila Zonzini Ramos, Nathalia Zocal Pereira dos Santos, Fábio Tadeu Arrojo Martins, Arthur Menino Castilho, Edi Lúcia Sartorato

**Affiliations:** 1 Human Molecular Genetics Laboratory, Molecular Biology and Genetic Engineering Center (CBMEG), University of Campinas (UNICAMP), Campinas, São Paulo Brazil; 2 ENT Department, Faculty of Medical Sciences, University of Campinas (UNICAMP), Campinas, São Paulo Brazil

**Keywords:** Hearing loss, Genetic testing, MassARRAY

## Abstract

**Background:**

Recent advances in molecular genetics have enabled to determine the genetic causes of non-syndromic hearing loss, and more than 100 genes have been related to the phenotype. Due to this extraordinary genetic heterogeneity, a large percentage of patients remain without any molecular diagnosis. This condition imply the need for new methodological strategies in order to detect a greater number of mutations in multiple genes. In this work, we optimized and tested a panel of 86 mutations in 17 different genes screened using a high-throughput genotyping technology to determine the molecular etiology of hearing loss.

**Methods:**

The technology used in this work was the MassARRAY iPLEX® platform. This technology uses silicon chips and DNA amplification products for accurate genotyping by mass spectrometry of previous reported mutations. The generated results were validated using conventional techniques, as direct sequencing, multiplex PCR and RFLP-PCR.

**Results:**

An initial genotyping of control subjects, showed failures in 20 % of the selected alterations. To optimize these results, the failed tests were re-designed and new primers were synthesized. Then, the specificity and sensitivity of the panel demonstrated values above 97 %. Additionally, a group of 180 individuals with NSHL without a molecular diagnosis was screened to test the diagnostic value of our panel, and mutations were identified in 30 % of the cases. In 20 % of the individuals, it was possible to explain the etiology of the HL. Mutations in *GJB2* gene were the most prevalent, followed by other mutations in in *SLC26A4*, *CDH23*, *MT-RNR1*, *MYO15A*, and *OTOF* genes.

**Conclusions:**

The MassARRAY technology has the potential for high-throughput identification of genetic variations. However, we demonstrated that optimization is required to increase the genotyping success and accuracy. The developed panel proved to be efficient and cost-effective, being suitable for applications involving the molecular diagnosis of hearing loss.

**Electronic supplementary material:**

The online version of this article (doi:10.1186/s12881-015-0232-8) contains supplementary material, which is available to authorized users.

## Background

Hearing loss (HL) is one of the most common sensory disorders, affecting around one in a thousand individuals, and can be caused by a variety of environmental and genetic factors [1]. In recent years, there have been significant advances in understanding the genetic causes of HL, which has assisted in disease diagnosis and other clinical practices.

Hereditary HL can be classified as syndromic or non-syndromic. The syndromic type is associated with distinctive clinical features and accounts for 30 % of congenital hereditary HL. Hearing loss that occurs in the absence of any other abnormal physical findings is known as non-syndromic hearing loss (NSHL) and accounts for the other 70 % [2–4].

More than 80 genes have been associated with NSHL [5–8]. Due to this high genetic heterogeneity, there is still great difficulty in determining the molecular etiology of HL. The most frequent genes associated with autosomal recessive inheritance that have been identified in NSHL are (in order of frequency) *GJB2, SLC26A4, MYO15A, OTOF, CDH23*, and *TMC1* [9, 10]. For each of these genes, at least 20 mutations have been reported. One specific mutation, c.35delG in the *GJB2* gene, is the most frequent in Caucasians, found in from 10 to 63 % of homozygous NSHL cases [11, 12]. None of the genes associated with non-syndromic autosomal dominant HL are very frequent, although the most common are *WFS1, KCNQ4, COCH*, and *GJB2* [9].

Identification of hearing impairment is extremely important because it allows positive cases to be properly referred for medical intervention and/or rehabilitation programs, and enables genetic counseling of the families affected. The extreme genetic heterogeneity of NSHL makes genetic diagnosis based on Sanger sequencing impractical, because this is a very expensive and time-consuming technique, besides not being feasible for genes with several exons. Thus, only a small number of genes are currently screened for determining the cause of HL, while a large percentage of patients remain without any genetic diagnosis. This indicates the need for new methodological strategies for detection of a greater number of mutations in multiple genes.

With the emergence of high-throughput technologies, this gap is being filled due the possibility to perform multiple simultaneous analyses, using small volumes of samples and reagents in the reactions, combining high accuracy with simplicity [13]. One of this promising technologies is the MassARRAY® platform (Sequenom Inc., San Diego, USA), that provides rapid measurement of DNA products, with modest multiplexing and minimal assay setup costs due to the use of unmodified oligonucleotide primers.

In order to address the genetic heterogeneity of HL and the labor and expense of conventional techniques, we developed a panel for diagnosis of non-syndromic hearing loss based on genotyping of 86 of the most frequent mutations that have already been described, using the MassARRAY® iPLEX mass spectrometry system. The power of the panel to detect mutations was demonstrated by applying it to 180 patients with presumed NSHL.

## Methods

### Patient recruitment and assessment

The study was carried out at the Human Molecular Genetics Laboratory of the Molecular Biology and Genetic Engineering Center (CBMEG) of the University of Campinas, in collaboration with the ENT department of the University of Campinas Teaching Hospital (São Paulo, Brazil). Blood samples were collected for genetic testing after obtaining written informed consent. The project was previously approved by the Research Ethics Committee of the Faculty of Medical Sciences of the University of Campinas (Report number 396/2006).

The technique was standardized using negative and positive control samples from 25 affected individuals for whom previous sequencing results were available for the *GJB2* gene and/or the *SLC26A4* gene, and for the m.1555A > G mitochondrial mutation in the *MT-RNR1* gene. Additionally, analysis was made of 180 unrelated Brazilian individuals with NSHL and without a molecular diagnosis. Inclusion criteria were: (1) bilateral HL, (2) no apparent syndromic features, (3) existence of audiometric and physical examinations, (4) a complete history to rule out obvious environmental causes of HL, and (5) onset of HL at age <50 years.

Obvious environmental causes of HL identified by the case history and physical examination included: prenatal factors (measles and CMV infections, and exposure to teratogenic agents during pregnancy), perinatal factors (anoxia and prematurity/care in a neonatal intensive care unit), postnatal factors (meningitis, otitis and head trauma), and other clinical features (otosclerosis, osteogenesis imperfecta, Usher syndrome, and Ménière’s disease).

For those patients with postlingual HL, hearing loss severity was defined according to pure-tone threshold hearing levels: mild (26–40 dB); moderate (41–70 dB); severe (71–90 dB); and profound (>90 dB) [14]. OAE, BERA, and infant audiometric testing were used to diagnose children with prelingual HL. All subjects were considered to have moderate to profound bilateral sensorioneural HL.

### Sample preparation

Genomic DNA was extracted from the leukocytes present in 4–8 mL of peripheral blood. The extraction was performed according to standard phenol-chloroform protocols. The purity and concentration of the samples were checked using a NanoDrop® 8000 spectrophotometer (Thermo Scientific) and a Qubit® 2.0 fluorometer (Invitrogen), respectively. Genomic DNA samples were diluted to obtain a final concentration of 10 ng/μL.

### The Sequenom MassARRAY® iPLEX platform

This technology uses silicon chips and DNA amplification products for accurate genotyping by mass spectrometry. Genomic DNAs are submitted to iPLEX Gold reaction and the product are transferred to chip wells by a robot. The genotypes are detected *in situ* by using matrix-assisted laser desorption ionization mass spectrometry (MALDI-TOF). This miniaturized method has the potential for accurate, high-throughput, low-cost identification of genetic variations [15].

**Fig. 1:**
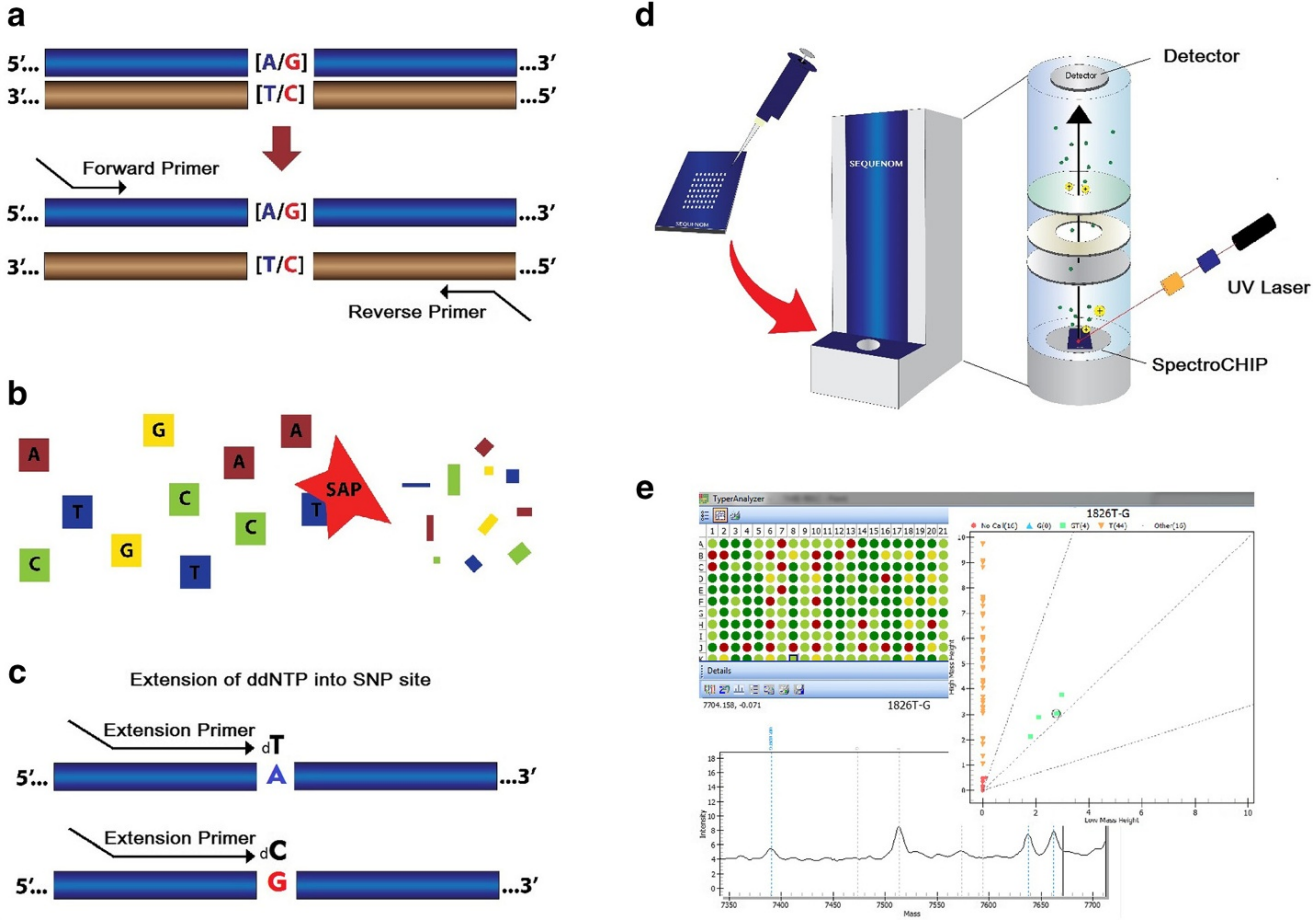
Steps in genotyping using MassARRAY® iPLEX System. A schematic of the genotype reaction of an A-to-G SNP. **a**. Locus-specific amplification reaction **b**. Treatment with SAP enzyme to neutralize unincorporated dNTPs. **c**. Locus-specific primer extension reaction (iPLEX assay). In this reaction, an oligonucleotide primer anneals immediately upstream of the polymorphic site being genotyped, the primer and amplified target DNA are incubated with mass-modified dideoxynucleotide terminators. The primer extension is made according to the sequence of the variant site. **d**. The products of the reactions are spotted on a spectroCHIP. The CHIP is placed into the mass spectrometer and each spot is then shot with a laser under vacuum by the matrix-assisted laser desorption ionization–time-of-flight (MALDI-TOF) method. A laser beam serves as desorption and ionization source in MALDI mass spectrometry. Once the sample molecules are vaporized and ionized, they are transferred electrostatically into a time-of-flight mass spectrometer (TOF-MS), where they individually detected. The mass of the extended primer is determined. **e**. Sequenom supplies software that automatically translates the mass of the observed primers into a genotype for each reaction

The iPLEX Gold technology consists of an initial locus-specific PCR reaction, followed by single base extension (SBE) using mass-modified dideoxynucleotide terminators of an oligonucleotide primer, which anneals immediately upstream of the polymorphic site of interest (Fig. 1) [15]. The product of these reactions are directly applied in a silicon chip. The mass of the extended primer is determined by means of MALDI-TOF mass spectrometry. The mass of the primer indicates the mutation of interest and the mass of added bases indicate the alleles present at the polymorphic site. Sequenom supplies a software (SpectroTYPER) that automatically translates the mass of the observed primers into a genotype for each reaction [16].

### Mutation selection and assay design

After a bibliographic search for genetic alterations related to HL in different populations, 94 alterations in 17 genes were selected for development of the panel (Table 1). The choice of genes to be studied was based on the most important genes involved in HL [9]. All these genes were described as affecting protein structures related to hearing, and were selected based on the frequency of reported mutations.

**Table 1:** Patterns of inheritance, genes and number of alterations selected for the panel

Inheritance	Gene	Locus	No. of selected alterations	OMIM	Transcripts IDs
Autosomal recessive	*GJB2*	DFNB1	22	121011	NM_004004
	*SLC26A4*	DFNB4	14	605646	NM_000441
	*MYO15A*	DFNB3	5	602666	NM_016239
	OTOF	DFNB9	16	603681	NM_194248
	*CDH23*	DFNB12	11	605516	NM_022124
	*TMC1*	DFNB7/11	4	606706	NM_138691
	*TMPRSS3*	DFNB8/10	5	605511	NM_024022
	*TRIOBP*	DFNB28	1	609761	NM_001039141
	*TMIE*	DFNB6	2	607237	NM_147196
	*DFNB59*	DFNB59	1	610219	NM_001042702
Autosomal dominant	*GJB2*	DFNA3	1	121011	NM_004004
	*WFS1*	DFNA6/14	1	606201	NM_006005.3
	*KCNQ4*	DFNA2	1	603537	NM_004700
	*COCH*	DFNA9	1	603196	NM_004086
	*TECTA*	DFNA8/12	4	602574	NM_005422
	*MIR-96*	DFNA50	2	613074	NR_029512
Mitochondrial	*MT-RNR1*	12S rRNA	2	561000	-
	*MT-TS1*	tRNASer(UCN)	1	590080	-

The alterations in the *GJB2* gene were selected based on files for about 2500 individuals with HL, who had been previously screened at Human Molecular genetics Laboratory at CBMEG (Campinas/Brazil). For other genes, mutations were selected based on how often they were reported in the literature, and only mutations that had been found in two or more families were included. Regarding the *OTOF, SLC26A4*, and *MYO15A* genes, all mutations that were identified in the Brazilian population were selected [17–19]. To check all selected alterations, see Additional file 1.

It was not possible to elaborate assays for the del(*GJB6*-D13S1830) and del(*GJB6*-D13S1854) deletions in the *GJB6* gene, because the MassARRRAY spectrometry technique presents a limitation to detect large delections/insertions.

The sequences covering the selected alterations were taken from the database of the National Center for Biotechnology Information [http://www.ncbi.nlm.nih.gov/] and Ensembl [http://www.ensembl.org/index.html]. Amplification and extension primers were designed using MassARRAY Assay Design v. 4.0 software. To avoid interaction among the primers, the software divided the PCR amplification and the Single Base Extension (SBE) into multiplex reactions. At the end of the standardization, 86 assays functioned properly, and the mutations were divided into 8 groups, containing 20, 18, 14, 9, 9, 8, 5 and 3 assays, respectively.

### Genotyping using the Sequenom MassARRAY® iPLEX platform

The whole process was performed according to the manufacturer’s instructions for the multiplex reactions, including the PCR amplification, the Shrimp Alkaline Phosphatase (SAP) treatment, and the primer extension reactions using the iPLEX Gold assay (Sequenom Inc., San Diego, USA). The reaction products were then dispensed onto a 384-SpectroCHIP using the MassARRAY nanodispenser and analyzed using the MassARRAY platform. Mass signals for the different alleles were captured with high accuracy by matrix-assisted laser desorption/ionization time-of-flight mass spectrometry (MALDI-TOF MS). Typer v. 4.0 (Sequenom Inc., San Diego, USA) was used to process the raw data obtained from the assays.

### Specificity and sensitivity calculations

The calculations were made individually for 38 alterations that had been previously detected in the control group. Specificity is considered the proportion of true negatives that are correctly identified by the test [20]. The calculation was made by the fraction of the genotypes who do not have mutations (True Negatives) to those who truly do not have mutations (True Negatives + False Positives).

$$ \mathrm{Specificity}=\frac{TN}{TN+FP} $$

Sensitivity is the proportion of true positives that are correctly identified by the test [20]. Sensitivity is a fraction of genotypes with mutations detected by the test (True Positives) among those who actually have mutations (True Positives + False Negatives)

$$ \mathrm{Sensitivity}=\frac{TP}{PT+FN} $$

### Validation of the results

All samples were tested for the mutations c.35delG and c.-23 + 1G > A (commonly known as IVS1 + 1G > A) in the *GJB2* gene, the mutations del(*GJB6*-D13S1830) and del(*GJB6*-D13S1854) in the *GJB6* gene, and the m.1555A > G mutation in the *MTRNR1* gene.

Allele-specific PCR was used to detect the c.35delG mutation, as described elsewhere [21]. *GJB2* mutations were screened by direct sequencing of the coding region of the gene [22, 23]. A multiplex PCR methodology was used to detect del(*GJB6*-D13S1830) and del(*GJB6*-D13S1854) mutations, according to the procedures reported previously [24, 25]. Analysis of m.1555A > G was performed by PCR amplification followed by digestion with the *Bsm*AI restriction endonuclease [26]. To validate mutations found in other genes, only the corresponding exon containing the mutation was sequenced.

## Results

### Optimization

In the first genotyping using mass spectrometry of 15 control patients in triplicate, the genotyping call was calculated to be 74 %. Additionally, in all the control samples, 19 out of 94 mutations presented a failure.

In an attempt to improve the results, and minimize the occurrence of primer interactions and incorrect primer annealing, we diminished the number of mutations to be screened in each multiplex reaction, and the maximum number of mutations was limited to 20 for each well. In addition, the extension primers of all the 19 reactions that failed were redesigned in reverse sequences using the MassARRAY Assay Design software. This resulted in a greater number of groups of multiplex reactions (eight rather than six) used to analyze all the 94 mutations in each patient.

After optimization, new screening of 25 individuals of the control group was performed indicating that the genotyping call rate of the technique increased to 91 % and the sensitivity and specificity of the panel exceeded 97 %. These calculations were made comparing results of 38 mutations previously analyzed by Sanger sequencing with those results obtained by MassARRAY (Table 2). Following primer redesign, of 19 assays that were not working, 11 assays started to function properly, but 8 assays still failed (Table 3). In seven of these assays, no allele was detected, and one assay (p.V153I in the *GJB2* gene) continued to show false negative results, even after redesigning the extension primer. These eight mutations were excluded from the panel and were not considered in subsequent analyses.

**Table 2:** Comparison of genotyping results of 38 alterations obtained by MassARRAY® iPLEX system before and after optimization of the panel

	TP	FP	TN	FN	GC	SP	SN
Before optimization (%)	3.2	3.1	93.23	0.47	74	97	87
After optimization (%)	4.04	1.96	93.89	0.12	91	98	97

*TP* True positives, *FP* False positives, *TN* True negatives, *FN* False negatives, *GC* Genotyping Call, *SP* Specificity, *SN* Sensitivity

**Table 3:** Mutations that failed and had the assays excluded of the panel

Gene	Alteration	Protein change
*GJB2*	c.283G > A	p.V95M
*GJB2*	c.457G > A	p.V153I
*MYO15A*	c.6796G > A	p.V2266M
*OTOF*	c.2122C > T	p.R708*
*TMPRSS3*	c.1221C > T	p.P404L
*TECTA*	c.3107G > A	p.C1036Y
*TMIE*	c.241C > T	p.R81C
*CDH23*	c.6133G > A	p.D2045N

*translation termination (stop) codon.

### Mutation screening

After standardizing the panel with 86 mutations, this set of variants was screened using the mass spectrometry system in a group of 180 individuals with moderate to profound non-syndromic HL. The technique identified genetic alterations in seven genes in a total of 54 individuals (Table 4). Mutations in the *GJB2* gene were found in 43 cases, with c.35delG being the most prevalent, identified in at least one allele in 28 individuals. The gene with the second largest number of mutations detected by mass spectrometry in our sample was *SLC26A4*, with p.V609G being the most frequent variant (detected in four cases). Mutations in the *MT-RNR1*, *MT-TS1*, *OTOF*, *MYO15A*, and *CDH23* genes were also identified.

**Table 4:** Molecular alterations detected in 180 individuals

No. of cases	Alterations detected	Gene(s)	Onset	Severity
20	c.35delG/c.35delG	*GJB2*	Prelingual	Severe/profound
3	p.V27I/wt	*GJB2*	Prelingual/postlingual	Moderade/profound
2	c.35delG/wt	*GJB2*	Postlingual	Moderade/profound
2	p.M34T/wt	*GJB2*	Prelingual/postlingual	Moderade/profound
2	p.V609G/wt	*SLC26A4*	Prelingual/postlingual	Severe/profound
2	m.1555A > G	*MT-RNR1*	Postlingual	Moderade/profound
1	c.35delG/wt; **Δ*GJB6*-D13S1830/**wt	*GJB2; GJB6*	Prelingual	Profound
1	c.35deG/p.L90P	*GJB2*	Postlingual	Moderade
1	c.35delG/c.-23 + 1G > A	*GJB2*	Prelingual	Profound
1	c.35delG/c.167delT	*GJB2*	Prelingual	Moderade-severe
1	c.35delG/p.W172*	*GJB2*	Prelingual	Profound
1	p.35delG/p.M34T	*GJB2*	Postlingual	Moderade
1	p.E47*/wt; **Δ*GJB6*-D13S1830/**wt	*GJB; GJB6*	Prelingual	Profound
1	p.K168R/wt	*GJB2*	Postlingual	Profound
1	p.M34T/p.V37I	*GJB2*	Postlingual	Moderade
1	p.L90P/wt	*GJB2*	Postlingual	Moderade
1	p.V37I/**p.V95M**	*GJB2*	Postlingual	Profound
1	p.R184P/c.35delG	*GJB2*	Prelingual	Profound
1	p.V27I/**p.E114G**	*GJB2*	Postlingual	Moderade
1	p.W24*/p.W24*	*GJB2*	Postlingual	Profound
1	p.R75Q/wt	*GJB2*	Postlingual	Moderade
1	p.V27I/wt; p.V609G/wt	*GJB2; SLC26A4*	Postlingual	Profound
1	p.V138F/wt	*SLC26A4*	Postlingual	Profound
1	p.V609G/wt; c.5800_5801dupC/wt	*SLC26A4; OTOF*	Postlingual	Profound
1	p.R776C/wt	*SLC26A4*	Postlingual	Profound
1	p.R1746Q/wt	*CDH23*	Postlingual	Moderade
1	m.7445A > G	*MT-TS1*	Prelingual	Profound
1	p.S3525G/wt	*MYO15A*	Prelingual	Profound
1	**Δ*GJB6*-D13S1854/**wt	*GJB6*	Postlingual	Moderade

*wt* wild type. *translation termination (stop) codon.  Alterations in bold were detected by conventional techniques

A comparison of the results obtained by mass spectrometry and conventional techniques is provided in Table 4, where the alterations shown in bold type were only detected by the conventional techniques because they were not included in the panel.

The c.10573delA mutation in *MYO15A* gene was identified in one patient by mass spectrometry. However, the Sanger sequencing revealed a different alteration at the same site, c.10573A > G (p.S3525G). This occurred because iPLEX MassARRAY® technique detects only one base after the extension primer binding region, and for both alterations the base was a guanine instead of an adenine. The c.10573delA mutation corresponds to the deletion of an adenine, and the next base in the sequence is a guanine. In the case of the p.S3525G mutation, a substitution of the adenine for a guanine occurs at the same site. The p.S3525G alteration has been reported as a single nucleotide variation (SNV), according to the National Center for Biotechnology Information [http://www.ncbi.nlm.nih.gov/], and no data of clinical significance was available. To assess the possible damaging effect of amino acid substitution, four different *in silico* software analyses were used: SIFT [http://sift.jcvi.org/]; PolyPhen [http://www.polyphen.com]; Align-GVGD [http://agvgd.iarc.fr/]; and Mutation Taster [http://www.mutationtaster.org]. In all analyses, the effect was damaging for the myosin XV protein.

The genotyping cost using the developed panel to screen 4128 reactions is US$ 950 per SpectroCHIP (Table 5). Then, the cost per reaction (SNP/mutation) is approximately US$ 0.23. Taking into consideration that our panel uses eight wells per patient, it is possible to genotype 48 patients in a single 384-SpectroCHIP. Therefore, the costs per patient, including materials and reagents, is approximately US$ 20. These values do not include the labor, equipment costs, maintenances, and operational expenditure.

**Table 5:** Costs in dollars to genotype a set of 86 alterations related to NSHL using the MassARRAY® iPLEX System

Materials/reagents	Cost per chip	Cost per patient
Chips and kits sequenom	US$ 800.00	US$ 16.7
Primers	US$ 100.00	US$ 2.08
Consumables	US$ 50.00	US$ 1.04
*Total*	US$ 950.00	US$ 19.8

## Discussion

The MassARRAY iPLEX technology has the potential for high-throughput identification of genetic variations; however, our results demonstrated that to increase the genotyping success and accuracy, the optimization is required. After redesigning the assays that failed in the initial tests and genotyping control samples, sensitivity of the technique raised from 87 % to 97 %, the specificity increases from 97 % to 98 % and genotyping call rate increases from 74 % to 91 %.

Studies that have screened multiple mutations simultaneously in a substantial number of patients are scarce in the literature. In this study, the etiology of NSHL was investigated in a sample of 180 Brazilian individuals. These patients were selected carefully, and those cases with possible environmental etiology or with clinical features not compatible with sensorineural NSHL, were excluded.

After screening by mass spectrometry, we identified mutations in 32 patients with prelingual HL, and 22 patients with postlingual HL, totaling 54 patients. 32 out of 54 patients, (59.3 %) had family history of HL. Among the 17 genes analyzed, mutations were identified in seven of them, five autosomal (*GJB2, SLC26A4, MYO15A, OTOF,* and *CDH23*) and two mitochondrial (*MT-RNR1* and *MT-TS1*). The results indicated a predominance of mutations in connexin 26 (*GJB2*), in concordance with previous works [9, 23–25, 27].

The c.35delG mutation in the *GJB2* gene was identified in 15.6 % of the patients, and in 11.1 % of cases, this mutation was found in homozygosis, explaining the etiology of deafness. All of the individuals that were homozygous for this mutation had profound prelingual HL. The molecular identification of the c.35delG is usually made by allele-specific PCR or PCR followed by enzymatic restriction. These reactions are relatively inexpensive and are recommended as the first genetic test performed.

Other mutations in the *GJB2* gene were identified by mass spectrometry in 20 of the analyzed patients. To detect *GJB2* mutations, the coding exon of this gene is usually sequenced. As Sanger sequencing is expensive and time consuming, mass spectrometry offers an useful alternative procedure.

Mutations in the *SLC26A4* gene are the second most frequent cause of NSHL [9]. We identified six individuals with three different mutations in this gene. The p.V609G variant was the most prevalent, found in four individuals (2.2 % of the sample population). Functional studies have demonstrated that there is a partial loss of protein activity when this alteration is present, indicating a potential pathogenic effect, especially if associated with other genetic or environmental factors [28]. In all the four individuals, the p.V609G was identified in only one allele of the gene, so the cause of HL was not elucidated. The *SLC26A4* gene has 21 exons, so it would be expensive to perform a routine sequencing of all of them. Therefore, genotyping the selected set of mutations with mass spectrometry could be a useful option for initial analysis of this gene.

The del(*GJB6*-D13S1830) and del(*GJB6*-D13S1854) deletions in the *GJB6* gene [24, 25], could not be included in our panel. However, these mutations were screened in all patients, as was previously described. The del(*GJB6*-D13S1854) deletion was found in one heterozygous individual, and compound heterozygous genotypes involving del(*GJB6*-D13S1830) and *GJB2* mutations were identified in two individuals.

Three other mutations: p.R1746Q, c.5800_5801dupC, and p.S3525G, were identified in heterozygous genotypes, in the genes *CDH23*, *OTOF*, and *MYO15A*, respectively. These mutations do not explain the etiology of HL because they segregate with autosomal recessive inheritance. In subsequent studies, it would be necessary to sequence these genes in an attempt to find mutations in the second allele that could explain the cause of HL in these individuals.

The p.S3525G (c.10573A > G) mutation in the *MYO15A* gene was identified here by Sanger sequencing. No significant clinical data were available for this alteration, so *in silico* analyses were performed using four different software packages in order to assess the possible damaging effect of amino acid substitution. All these analyses indicated protein damage as an outcome. It is possible that this alteration contributes to hearing loss if it is present in both alleles or associated with other mutation in *MYO15A.*

Two mutations in mitochondrial genes were identified: m.1555A > G in *MT-RNR1*, detected in two individuals, and m.7445A > G in *MT-TS1*, detected in one case. According to previous studies, m.1555A > G is a common cause of genetic HL in Brazil. It was found in approximately 2 % of unselected cases of HL, and it was recommended for the inclusion in molecular diagnostic testing for HL [29, 30]. Early identification of patients with HL due to mutations in mitochondrial DNA can influence genetic counseling regarding maternal inheritance, enable avoidance of known risk factors, and assist pharmacological strategies for the prevention or reduction of HL progression [31].

Taking into account our data and the need to minimize costs and time, a pipeline trial is recommended before proceeding with mutations analysis using high-throughput methods. The first step should be to evaluate the clinical history of the patient in order to exclude obvious environmental etiologies. The second one is to assess the age of onset and the degree of HL. In cases of prelingual HL, it is recommend screening for the c.35delG mutation and *GJB6* deletions by conventional techniques, due to the high frequency of these mutations as a cause of prelingual HL. For the remaining cases where molecular diagnosis is unclear, as well as cases of postlingual HL, the screening for the main mutations involved in HL by high throughput or conventional methods is recommended.

The genetic evaluation identified mutations in 30 % of the analyzed patients, and emphasized the importance of molecular testing, however in only 20 % of the cases the etiology of HL was concluded. The high number of cases with unknown etiology was possibly due to the broad genetic heterogeneity involved in NSHL, and also to the selection of mutations made to this study, since this selection was based on studies of other populations, including countries with high rates of consanguineous marriages, and might not be representative of the Brazilian population.

The continuous advances in DNA sequencing technologies allowed us to identify genetic alterations in a growing number of individuals. Therefore, they enabled the identification of the most frequent mutations in different populations. With this knowledge, the screening of specific mutations by high-throughput technologies could be really interesting to use in routine tests, since panels with specific assays could be customized for each population, reducing costs and enabling the rapid screening of a large number of patients.

There are several reliable, relatively simple and inexpensive methods currently in use to detecting mutations. Recently, array-based tests have been developed for NSHL, using different genotyping technologies: APEX microarray [32], designed Affymetrix resequencing microarrays [33] and TaqMan® OpenArray™ [34]. All these technologies have in common the use of oligonucleotides applied on a solid surface and fluorescent compounds. The OpenArray and APEX array focus specifically on the detection of previously reported mutations, whereas the Affymetrix resequencing array allows for the discovery of new mutations. The APEX microarray allows some versatility, since it is possible add SNPs after ordering the custom test. In contrast, OpenArray and resequencing arrays do not allow for modifications after achievement of the plates/chips. With reference to costs, the screening of mutations with Affymetrix resequencing arrays are quite high in comparison with the others, making it unfeasible the use of this technique in a routine laboratory setting [35, 36].

In contrast to these technologies, the MassARRAY iPLEX does not uses fluorescent compounds, and analyses direct DNA products. As the OpenArray and APEX, this technique has the limitation of detect only previously reported mutations. However, the great advantage of MassARRAY iPLEX is the versatility and flexibility, since the assays are not pre-spotted into the chip by the manufacturer. Therefore, you can easily add or remove tests and genotyping different sets of mutations on the same chip. Moreover, the number of wells analyzed at a time can be variable, since the same chip can be used in different experiments.

All the techniques mentioned above, including MassARRAY, need be confirmed by other techniques when mutations were found. Our results demonstrated that even after optimization, false positive results were observed in 1.96 % of the genotypes. Therefore, the validation of positive cases must be carried out to ensure the reliability of the results.

Sanger sequencing is considered the golden standard for mutation identification due to its accuracy. However, Sanger sequencing is labor intensive and has high per-base sequencing costs. It can be effectively only if used to screen genes with a limited number of exons, or to confirm mutations found with other technologies.

Next generation sequencing is the most efficient method to identify mutations, especially in heterogeneous disorders such as hereditary hearing loss. Recent developments in NGS and DNA capture technologies provide a potential approach for accurate molecular diagnosis of HL [37–39]. The main advantage of NGS over current diagnostic methods is that a significantly higher rate of successful diagnosis can be obtained by screening both known and novel mutations in all HL genes simultaneously. However, its high cost and delay in data analysis make it impractical for application in the diagnosis of many individuals.

The high-throughput techniques such as IPLEX MassARRAY® system is a good option for initial screening of the most frequent mutations, since it enables genotyping a substantial set of known alterations simultaneously, in shorter periods of time, and at lower cost, compared to the screening of the main mutations performed with conventional techniques like PCR, Sanger sequencing and enzymatic restriction. The cost of conventional routine screening of mutations in the *GJB2, GJB6*, and *MT-RNR1* genes for diagnosis of hearing loss is approximately US$ 30 per patient. In contrast, the cost of mass spectrometry screening of 86 genetic mutations in nine genes is approximately US$ 20 per patient, evidencing, this way, a better cost-benefit. This comparison only include the costs of reagents and materials used in our research laboratory.

## Conclusions

We have developed and tested a panel based on MassARRAY technology for screening 86 mutations related to NSHL. This technology has the potential for high-throughput identification of genetic variations; however, we demonstrated that to increase the genotyping success and accuracy, optimization is required. The panel proved to be cost-effective and efficient, due to the high values obtained in sensitivity and specificity. We tested the panel in affected Brazilian individuals with NSHL without a molecular diagnosis. Genetic alterations were identified in 30 % of them, and the etiology of NSHL was concluded in 20 % of the cases. These results demonstrate that the developed panel is suitable and helpful to be applied in the diagnosis of hearing loss.

## Additional file

Additional file 1: **Patterns of inheritance, mutations selected for the study, and their respective genes and references.** Table with all alterations selected for the panel and references. [42-92] (DOCX 20 kb)

## Abbreviations

BERA: Brainstem evoked response audiometry
CMV: Cytomegalovirus
HL: Hearing loss
NSHL: Non-syndromic hearing loss
OAE: Otoacoustic emissions
PCR: Polymerase chain reaction
RFLP: Restriction fragment length polymorphism
SBE: Single base extension
SNP: Single nucleotide polymorphism
SNV: Single nucleotide variation
US$: United States dollar
