# Supplementary material for: Screening of genetic alterations related to non-syndromic hearing loss using MassARRAY iPLEX® technology
Source: BMC Med Genet. 2015 Sep 23;16:85. doi: 10.1186/s12881-015-0232-8 (PMC4581412; doi:10.1186/s12881-015-0232-8)
Supplement: Additional file 1: — Patterns of inheritance, mutations selected for the study, and their respective genes and references. Table with all alterations selected for the panel and references. [42-92] (DOCX 20 kb) [file 12881_2015_232_MOESM1_ESM.docx]

# Additional file

### **Additional file 1- Patterns of inheritance, mutations selected for the study, and their respective genes and references.**

| **Inheritance** | **Gene** | **Alteration** | **Protein change** | **References** |
| --- | --- | --- | --- | --- |
| Autos. Recessive | *GJB2* | c. -23+1G>A | Splicing defect | [40] |
|  |  | c.35delG | G12Vfs | [41] |
|  |  | c.71>A | p.W24* | [23] |
|  |  | c.79G>A | p.V27I | [42] |
|  |  | c.101T>C | p.M34T | [24] |
|  |  | c.109G>A | p.V37I | [42] |
|  |  | c.139G>T | p.E47* | [22] |
|  |  | c.167delT | p.L56Rfs | [41] |
|  |  | c.235delC | p.L79Cfs | [43] |
|  |  | c.269T>C | p.L90P | [44] |
|  |  | c.279G>A | p.M93I | [45] |
|  |  | **c.283G>A** | **p.V95M** | **[42]** |
|  |  | c.339T>G | p.S113R | [42] |
|  |  | c.385G>A | p.E129K | [46] |
|  |  | c.427C>T | p.R143W | [47] |
|  |  | c.439G>A | p.E147K | [48] |
|  |  | **c.457G>A** | **p.V153I** | **[49]** |
|  |  | c.503A>G | p.K168R | [50] |
|  |  | c.516G>A | p.W172* | [51] |
|  |  | c.550C>G | p.R184W | [52] |
|  |  | c.551G>C | p.R184P | [23] |
|  |  | c.617A>G | p.N206S | [46] |
|  | *SLC26A4* | c.279delT | p.S93Rfs | [53] |
|  |  | c.412G>T | p.V138F | [54] |
|  |  | c.425C>T | p.P142L | [18] |
|  |  | c.446G>A | p.G149R | [55] |
|  |  | c.578C>T | p.T193I | [56] |
|  |  | c.845G>A | p.C282Y | [18] |
|  |  | c.1001+1G>A | Splicing defect | [57] |
|  |  | c.1226G>A | p.R409H | [54] |
|  |  | c.1229C>T | p.T410M | [57] |
|  |  | c.1238A>G | p.Q413R | [58] |
|  |  | c.1334T>G | p.L445W | [54] |
|  |  | c.1707+5G>A | Splicing defect | [55] |
|  |  | c.1826T>G | p.V609G | [59] |
|  |  | c.2326C>T | p.R776C | [59] |
|  | *MYO15A* | c.3313G>T | p.E1105* | [60] |
|  |  | c.3336delG | p.R1113Vfs | [60] |
|  |  | **c.6796G>A** | **p.V2266M** | **[60]** |
|  |  | c.9957_9960delTGAC | p.D3320Tfs | [19] |
|  |  | c.10573delA | p.S3525Afs | [19] |
|  | *OTOF* | c.1552_1567delCGCAAGATTTCTAATG | p.R518Tfs | [17] |
|  |  | c.1601delC | p.P534Qfs | [61] |
|  |  | c.1841G>A | p.G614E | [17] |
|  |  | **c.2122C>T** | **p.R708*** | **[62]** |
|  |  | c.2348delG | p.G783Afs | [63] |
|  |  | c.2485C>T | p.Q829* | [64] |
|  |  | c.2905_2923delinsCTCCGAGCGCA | p.A969Pfs | [61] |
|  |  | c. 3239G>C | p.R1080P | [17] |
|  |  | c.3400C>T | p.R1134* | [61] |
|  |  | c.3413T>C | p.L1138P | [61] |
|  |  | c.4227-1G>T | Splicing defect | [61] |
|  |  | c.4491T>A | p.Y1497* | [65] |
|  |  | c.4960G>A | p.G1654S | [17] |
|  |  | c.5431A>T | p.K1811* | [17] |
|  |  | c.5785A>C | p.N1929H | [17] |
|  |  | c.5800_5801dupC | p.L1934Pfs | [61] |
|  | *CDH23* | c.719C>T | p.P240L | [66] |
|  |  | c.902G>A | p.R301Q | [66] |
|  |  | c.2968G>A | p.D990N | [67] |
|  |  | c.4756G>C | p.A1586P | [68] |
|  |  | c.5147A>C | p.Q1716P | [66] |
|  |  | c.5237G>A | p.R1746Q | [69] |
|  |  | c.6604G>A | p.D2202N | [67] |
|  |  | c.6050-9G>A | Splicing defect | [70] |
|  |  | c.6085C>T | p.R2029W | [66] |
|  |  | **c.6133G>A** | **p.D2045N** | **[67]** |
|  |  | c.6442G>A | p.D2148N | [68] |
|  | *TMC1* | c.100C>T | p.R34* | [71] |
|  |  | c.1165C>T | p.R389* | [72] |
|  |  | c.1334G>A | p.R445H | [73] |
|  |  | c.1939 T>C | p.S647P | [74] |
|  | *TMPRSS3* | c.207delC | p.T70fs | [75] |
|  |  | c.323-6G>A | Splicing defect | [76] |
|  |  | c.413C>A | p.A138E | [10] |
|  |  | c.916G>A | p.A306T | [77] |
|  |  | **c.1221C>T** | **p.P404L** | **[78]** |
|  | *TRIOBP* | c.1039C>T | p.R347* | [79] |
|  | *TMIE* | **c.241C>T** | **p.R81C** | **[80]** |
|  |  | c.250C>T | p.R84W | [80] |
|  | *DFNB59* | c.547C>T | p.R183W | [81] |
| Autos. Dominant | *GJB2* | c.224G>A | p.R75Q | [82] |
|  | *WFS1* | c.2146G>A | p.A716T | [83] |
|  | *KCNQ4* | c.827G>C | p.W276S | [84] |
|  | *COCH* | c.151C>T | p.P51S | [85] |
|  | *TECTA* | **c.3107G>A** | **p.C1036Y** | **[86]** |
|  |  | c.5509T>G | p.C1837G | [87] |
|  |  | c.5597C>T | p.T1866M | [88] |
|  |  | c.5668C>T | p.R1890C | [89] |
|  | *miR-96* | +13G>A | NA | [90] |
|  |  | +14C>A | NA | [90] |
| Mitochondrial | *MT-TS1* | m.7445A>G | NA | [91] |
|  | *MT-RNR1* | m.1494C>T | NA | [26] |
|  |  | m.1555A>G | NA | [92] |

*stop codon. Alterations in bold were not included in the panel.
